# Tick-Borne Encephalitis Virus Prevalence in Sheep, Wild Boar and Ticks in Belgium

**DOI:** 10.3390/v14112362

**Published:** 2022-10-26

**Authors:** Nadjah Radia Adjadj, Muriel Vervaeke, Charlotte Sohier, Mickaël Cargnel, Nick De Regge

**Affiliations:** 1 Unit of Exotic Viruses and Vector-Borne Diseases, Sciensano, 1180 Brussels, Belgium; 2 Agency of Nature and Forests, 1000 Brussels, Belgium; 3 Unit of Veterinary Epidemiology, Sciensano, 1050 Brussels, Belgium

**Keywords:** prevalence, sheep, wild boar, tick-borne encephalitis, ticks, PRNT, Belgium

## Abstract

Tick-borne encephalitis virus (TBEV) is the most important tick-borne zoonotic virus in Europe. In Belgium, antibodies to TBEV have already been detected in wildlife and domestic animals, but up-to-date prevalence data for TBEV are lacking, and no studies have assessed its seroprevalence in sheep. Serum samples of 480 sheep from all over Belgium and 831 wild boar hunted in Flanders (northern Belgium) were therefore screened for TBEV antibodies by ELISA and plaque reduction neutralization test (PRNT), respectively. The specificity of positive samples was assessed by PRNTs for TBEV and the Louping Ill, West Nile, and Usutu viruses. TBEV seroprevalence was 0.42% (2/480, CI 95%: 0.11–1.51) in sheep and 9.27% (77/831, CI 95%: 7.48–11.43) in wild boar. TBEV seroprevalence in wild boar from the province of Flemish Brabant was significantly higher (22.38%, 15/67) compared to Limburg (7.74%, 34/439) and Antwerp (8.61%, 28/325). Oud-Heverlee was the hunting area harboring the highest TBEV seroprevalence (33.33%, 11/33). In an attempt to obtain a Belgian TBEV isolate, 1983 ticks collected in areas showing the highest TBEV seroprevalence in wild boars were tested by real-time qPCR. No TBEV-RNA-positive tick was detected. The results of this study suggest an increase in TBEV prevalence over the last decade and highlight the need for One-Health surveillance in Belgium.

## 1. Introduction

Tick-borne encephalitis virus (TBEV) is an important viral tick-borne zoonosis in Europe and Asia, and its incidence seems to have increased in recent years [1,2,3]. This zoonotic neurotropic flavivirus is currently divided into at least five subtypes, namely the European (TBEV-Eu), Far-Eastern (TBEV-FE), Siberian (TBEV-Sib), Himalayan (TBEV-Him), and recently detected Baikalian (TBEV-Bkl) subtypes [4,5,6]. These subtypes differ not only in their geographical distribution but also in their clinical manifestations and the severity of the disease [7,8]. TBEV is mostly transmitted by the tick *Ixodes ricinus* in Western Europe and *I. persulcatus* in Eastern Europe and Siberia, but alimentary transmission via the raw milk or raw milk products of viremic ruminants can also occur [9,10,11,12,13]. At least 11 European countries have already reported alimentary TBEV infections and outbreaks, with recent examples from Croatia in 2019 and France in 2020 [14,15,16]. TBEV is distributed within the tick population mainly through cofeeding and trans-stadial transmission, and more rarely through trans-ovarial transmission [6,17]. TBEV prevalence in questing ticks is rather low (<1%), and its circulation between ticks and hosts is geographically restricted to small foci [3,18,19,20]. Rodents constitute the main reservoir hosts of TBEV, but different species of wild and domestic mammals (particularly deer, wild boar, sheep, cattle, and goats) play an indirect role in TBEV circulation by hosting and enabling tick multiplication [6,21]. Although humans are incidental and dead-end hosts, about 3411 cases were reported in 2019 in Europe from 25 countries [22]. About 2/3 of human TBEV infections are asymptomatic, but in some cases TBEV can cause encephalitis with serious sequelae [23,24]. Symptomatic disease is often characterized by a biphasic pattern with flu-like symptoms in the first stage, an asymptomatic interval, and a second stage with neurological manifestations ranging from mild meningitis to severe encephalitis with or without myelitis and spinal paralysis [25,26]. In animals, TBEV infection is mostly asymptomatic, but rare clinical cases with neurological symptoms have been reported [27,28,29]. Infected ruminants are viremic for a short period but develop long-lasting antibodies; thus, sheep and goats have recently been used for the monitoring of TBEV in different countries [3,30,31,32,33]. In addition, sheep and goats are easy to sample and are exposed to ticks by grazing in the meadows, which makes them optimal sentinels for the detection of TBEV [3,33,34]. Wild boar have also been used as sentinels for TBEV surveillance given their preference for landscapes that are also suitable habitats for *I. ricinus* tick populations and the formation of natural TBEV foci [35].

Serological tests are the most common methods for TBEV diagnosis and epidemiological studies, since the short viremic phase of infection limits the usefulness of molecular assays, which are also expensive and time-consuming. Serological testing often consists of an initial screen via an enzyme-linked immunosorbent assay (ELISA), followed by the confirmation of borderline and positive results with the gold-standard seroneutralization test (SNT). Both ELISA and SNT have previously been used to detect TBEV-specific antibodies in animals including sheep and wild boar. However, serological tests suffer some drawbacks, such as cross-reaction with antibodies against other flaviviruses [33,34,36].

In Belgium, antibodies against TBEV were detected in cattle (2.61–4.29%), wild boar (4.20%), roe deer (5.1%), and dogs (0.11%) between 2011 and 2016 [37,38,39,40,41]. Three confirmed autochthonous human cases were also recently reported [42]. However, no detection in ticks or the isolation of the virus itself have been reported, and the most recently published seroprevalence data date back to 2016. The aim of this study was to provide up-to-date prevalence data for TBEV in Belgium. Therefore, this study assessed the country-wide prevalence of TBEV in sheep and the prevalence in wild boar serum and questing ticks from Flanders.

## 2. Materials and Methods

### 2.1. Samples

#### 2.1.1. Sheep Sera

The sheep sample size (*n* = 480) necessary to estimate the seroprevalence in Belgium with 95% confidence and a precision of 2% was determined using the Epitools website (https://epitools.ausvet.com.au/) (accessed on 10 February 2020). We therefore considered a design prevalence of 4%, based on the expected prevalence provided by the literature [39], together with a test sensitivity of 97% and a test specificity of 99%, as reported by the manufacturer of the selected ELISA kit. Sheep samples (*n* = 480) were selected from the already available samples (*n* = 7299) collected during the “Maedi-Visna (MVV) and Caprine Arthritis and Encephalitis (CAE) screening program 2019”. This is a voluntary program set up by the federal government (Royal Decree 24-03-1993 for MVV and 27-11-1997 CAE) that allows the acquisition of a free small-ruminant lentivirus (SRLV) certificate.

**Figure 1:**
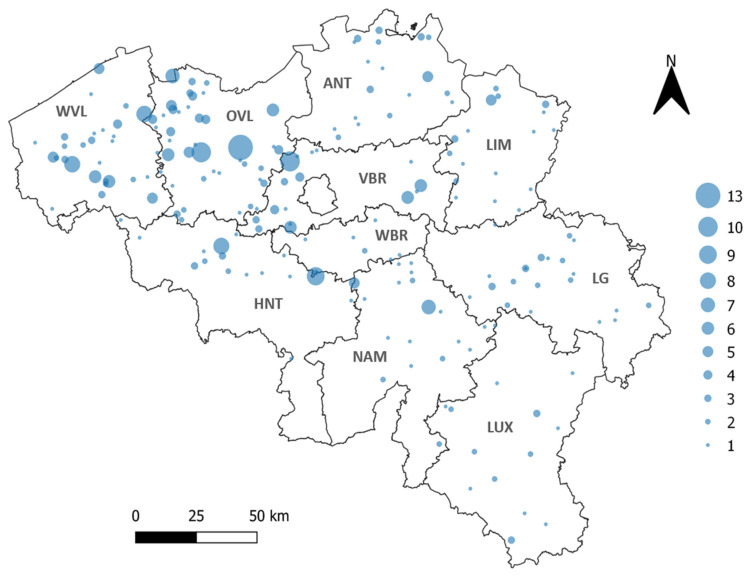
Geographical repartition of sheep samples (indicated with blue circles). The 10 Belgian provinces are indicated in bold. WVL: West Flanders, OVL: East Flanders, ANT: Antwerp, LIM: Limburg, VBR: Flemish Brabant, WBR: Walloon Brabant, HNT: Hainaut, NAM: Namur, LG: Liege, LUX: Luxembourg. The size of the blue circle corresponds to the number of samples per farm (from 1 to 13 samples).

In order to obtain the country-wide seroprevalence, a randomized sample selection process stratified proportional to the number of sheep herds per province (obtained from [43]) was performed. The number of samples selected from each province can be found in Table 1. All Belgian provinces (*n* = 10) were included in this nation-wide screening (Figure 1). 

#### 2.1.2. Wild Boar Sera

**Figure 2:**
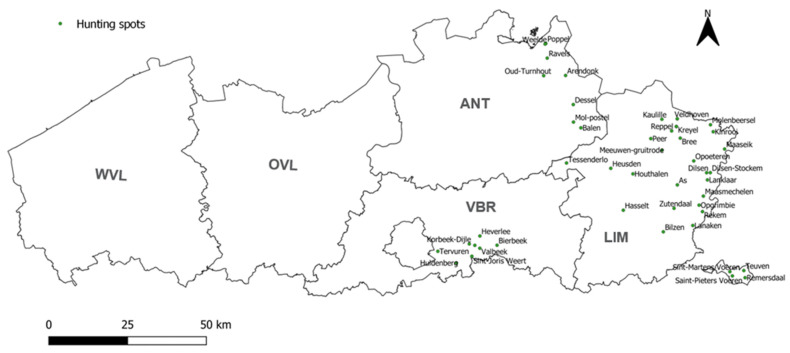
Geographical repartition of the 50 wild boar hunting spots where sera were collected between May 2019 and October 2020. The 5 Flemish provinces are indicated in bold. WVL: West Flanders, OVL: East Flanders, ANT: Antwerp, LIM: Limburg, VBR: Flemish Brabant. The 4 isolated spots in the bottom right (Teuven, Remersdaal, Saint-Pieters Voeren, and Sint-Martens Voeren) belong to the province of Limburg.

All serum samples were collected from wild boar hunted in northern Belgium from May 2019 to October 2020 in the framework of the active monitoring of classical and African swine fever virus, brucellosis, tuberculosis, and Aujeszky’s disease by the Flemish government. All wild boar sera samples with sufficient volumes (*n* = 831) from 50 different hunting locations within the provinces of Antwerp (*n* = 325), Limburg (*n* = 439), and Flemish Brabant (*n* = 67) were tested (Figure 2). These provide a dataset that corresponded to the geographical distribution of wild boar in Flanders [44]. No wild boar were hunted in the provinces of East Flanders (OVL) and West Flanders (WVL).

### 2.2. Ethics Statement

No specific ethical approval had to be obtained for this study, since the collection of blood from sheep and wild boar by a veterinarian is considered a routine veterinary practice and needs no specific approval from an ethical committee under current European and Belgian legislation (Directive 2010/63/EU of the European parliament and of the council of 22 September 2010 on the protection of animals used for scientific purposes; Belgian Royal Decree of May 2013 relating to the accommodation and care of experimental animals (C 2013/24221, chap I. §4)).

### 2.3. Serological Analysis

The presence of antibodies against TBEV in sheep sera was assessed using a commercial ELISA kit (IMMUNOZYM FSME IgG ALL SPECIES, Progen Biotechnik GmbH, Heidelberg, Germany) following the manufacturer’s instructions. The measured optical density (OD) was converted into Vienna units (VIEU/mL) based on the values of the calibrators included in the kit. Samples with more than 126 VIEU/mL were considered positive, samples with less than 63 VIEU/mL were considered negative, and samples with 63 to 126 VIEU/mL were considered borderline.

ELISA positive and borderline samples were assessed using the plaque reduction neutralization test (PRNT) to confirm the TBEV status and to evaluate potential cross-reactions with other flaviviruses, namely louping ill virus (LIV), West Nile virus (WNV), and Usutu virus (USUV). For this purpose, sheep sera were decomplemented (30 min at 56 °C), five-fold diluted, and then serially two-fold diluted (from 1/5 to 1/640) in Dulbecco’s Modified Eagle Medium (DMEM, Gibco^TM^ Thermo Fisher Scientific, Waltham, MA, USA) in 96-well plates. Subsequently, approximately 20 to 50 plaque-forming units of the viruses were added to each serum dilution and incubated for 1 h at 37 °C. The strains TBEV Neudörfl, LIV LI/31, Israeli WNV IS-98-ST1, and USUV Italy 2012 were used. Next, the virus–serum mixes were added to a 95% confluent monolayer of vero cells and incubated for 24–72 h at 37 °C, depending on the tested virus. Cells were then washed with PBS and fixed with methanol for immunofluorescence staining. Primary virus-specific mouse monoclonal anti-NS1 antibodies (R&D systems, Bio-Techne Ltd., Minneapolis, MN, USA) and a secondary Alexa fluor-conjugated goat anti-mouse IgG antibody (Invitrogen, Thermofisher Scientific, Waltham, MA, USA) were used. All sera were tested in duplicate, and the number of plaques per well was counted under the fluorescence microscope. Wells in which the number of plaques was reduced by 50% or more compared to the number of plaques in control wells were considered neutralized. Based on the literature [33,45,46,47,48], the cut-off for positivity was fixed at a titer of 1/10.

The presence of anti-TBEV antibodies in wild boar serum was directly assessed by PRNT. Given their suboptimal quality, wild boar sera were kaolin-treated beforehand, as previously described [49]. The samples were then decomplemented and tested in TBEV–PRNT following the aforementioned protocol. Positive samples further underwent PRNTs for LIV, WNV, and USUV to evaluate potential cross-reactions with other flaviviruses. Each sample received a final status based on the PRNT in which it reached the highest titer. When no 4-fold difference was found between PRNT titers for different flaviviruses, the samples received the status of flavivirus-positive.

### 2.4. Tick Collection

A total of 1983 (1515 nymphs, 216 females, 241 males, and 11 larvae) questing ticks were collected in June and October 2021 by flagging in areas with the top 5 highest TBEV prevalence in wild boar (in this study). These included 7 collection sites in total (encompassing wild boar hunting posts) within the forests of Hoge Vijvers Bos, Bladelse Heide, Kommiezenheide, and Luyksgestelse; the national park of Hoge Kempen; and the woodland of Meerdaalwoud. These areas are located in the provinces of Antwerp, Limburg, and Flemish Brabant within the northern part of Belgium (Flanders). The latter is characterized by a highly fragmented landscape and is one of the most densely populated regions in Europe. Forests cover about 11% of the total area in Flanders and are mainly composed of pine and poplar plantations and to a lesser extent oak, mixed deciduous, and beech forests [44,50]. The collection sites presented various habitats, such as forests, grasslands, fields, wetlands, and low shrubby vegetation including heather, all often interspersed by road networks. Collected ticks were morphologically identified to species level using a taxonomic key [51].

### 2.5. Tick Pooling and Homogenization, RNA Extraction, and qPCR Analysis

Ticks were pooled according to development stage, sex, and sampling site, so that each pool contained a maximum of 6 ticks. The 11 larvae were pooled all together. A total of 408 tick pools were prepared and homogenized in 500 µL of Minimum Essential Medium (MEM, Gibco^TM^ Thermo Fisher Scientific, Waltham, MA, USA) using 2 stainless steel beads (5 mm, Qiagen^®^) and a tissue lyser (TissueLyser II, QIAGEN^®^) for 5 min at 25 Hz. After centrifugation for 1 min at 10,000 rpm, 200 µL of tick homogenate was harvested for RNA extraction. RNA from tick homogenates was extracted using the IndiMag^®^ Pathogen Kit (Indical Bioscience, Leipzig, Germany) following the manufacturer’s protocol. Five microliters of eluted RNA (from individual extracts) was used to assess the presence of TBEV RNA by qPCR, as described by [36]. All samples were also tested for the presence of *Ixodes ricinus* β-actin as an extraction control. In each run, negative extraction and negative and positive amplification controls were also included. All qPCRs were carried out on a LightCycler 480 Real-Time PCR system (Roche, Basel, Switzerland) using the AgPath-ID™ One-Step RT-PCR Reagents (Applied Biosystems^TM^, Thermo Fisher Scientific, Waltham, MA, USA).

### 2.6. Statistical Analysis and Mapping

The TBEV prevalence and the 95% confidence interval (CI) were calculated using Wilson’s method implemented on the Epitools website (https://epitools.ausvet.com.au/) (accessed on 15 September 2022). Fisher’s exact tests or chi-square tests were used to compare the seroprevalence of TBEV in wild boar between the Belgian provinces. Statistical analyses were performed using GraphPad Prism 9. *p*-values < 0.05 were regarded as statistically significant. The maps of the geographical repartition of samples and hunting spots were made in QGIS^®^3.4 (Switzerland) using Belgium and Flanders vector layers in the Belgian Lambert 2005 EPSG projection.

## 3. Results

### 3.1. TBEV-Specific Antibody Detection in Sheep

**Figure 3:**
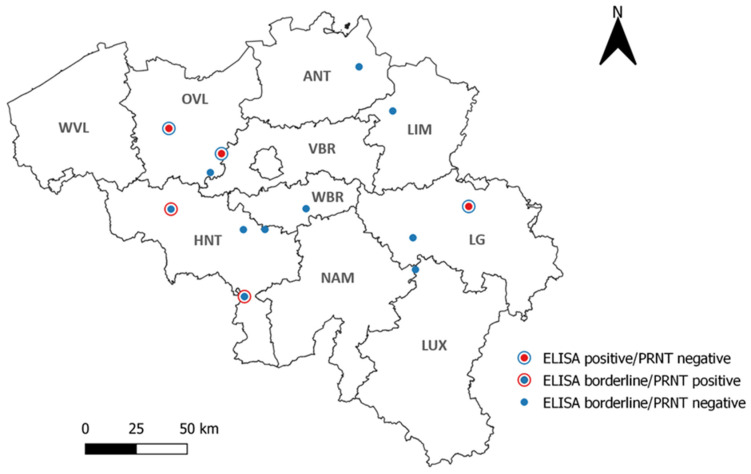
Geographical repartition of the 13 (out of 480) TBEV-positive and borderline sheep serum samples according to ELISA. WVL: West Flanders, OVL: East Flanders, ANT: Antwerp, LIM: Limburg, VBR: Flemish Brabant, WBR: Walloon Brabant, HNT: Hainaut, NAM: Namur, LG: Liege, LUX: Luxembourg.

The ELISA screening of the 480 sheep sera from all over Belgium resulted in three positive (0.63%) and ten borderline samples (2.08%). Two out of the three positives originated from East Flanders, while the third positive came from the province of Liege. Borderline samples, on the other hand, were distributed as follows: one borderline sample in the provinces of Luxembourg, Liege, Limburg, Walloon Brabant, Antwerp, and East Flanders; and four borderline samples in the province of Hainaut. No positive or borderline samples were found in the provinces of West Flanders and Flemish Brabant (Figure 3).

The ELISA TBEV-positive and borderline samples were further subjected to PRNTs for TBEV, LIV, WNV, and USUV in order to confirm their status and to check for flaviviral cross-reactivity. The three ELISA TBEV-positive samples tested negative in all four PRNTs. Out of the 10 ELISA TBEV borderline samples, two samples were confirmed to be TBEV-positive (at 1:20 titers), while two others were found to be USUV-positive (Figure 3). With only two confirmed TBEV-positive samples, the national TBEV seroprevalence in sheep was 0.42% (2/480, CI 95%: 0.11–1.51).

### 3.2. TBEV-Specific Antibody Detection in Wild Boar

**Figure 4:**
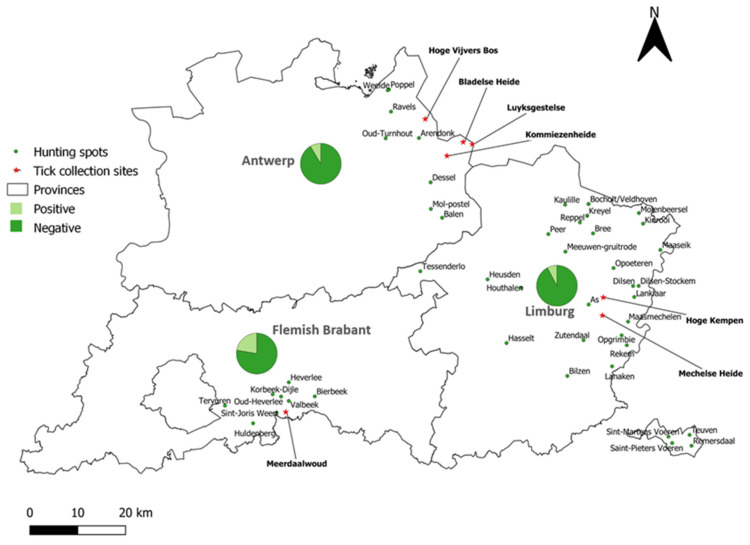
TBEV seroprevalence in wild boar sera per province. The hunting spots are indicated by green dots. The pie chart depicts the percentage of positive and negative samples per province. Tick collection sites are indicated with a red star.

Sera from 831 hunted wild boar were screened by PRNT for the presence of anti-TBEV antibodies. One hundred and forty-two samples were found to be positive (17.09%). These positive samples were further subjected to PRNTs for LIV, WNV, and USUV to evaluate potential cross-reactions with other flaviviruses. Based on the obtained neutralization titers in the different PRNTs, 77 of the 142 (54.23%) samples were classified as TBEV-positive, 28 (19.71%) as USUV-positive, and 37 (26.06%) as flavivirus-positive. Therefore, the minimal national TBEV prevalence was 9.27% (77/831, CI 95%: 7.48–11.43). Of the confirmed TBEV-positive samples, 27.27% (21/77) had a titer of 1:10, 28.57% (22/77) had a titer of 1:20, 16.88% (13/77) had a titer of 1:40, and 27.27% (21/77) reached a titer of 1:80. At the provincial level (Figure 4, Table 2), a significantly higher TBEV seroprevalence (chi^2^ test: *p* = 0.0005) was obtained in Flemish Brabant (22.39%) compared to Limburg (7.74%) and Antwerp (8.62%).

The analysis of TBEV prevalence per hunting area showed that TBEV-seropositive wild boar were found in 24 out of the 50 hunting spots. Oud-Heverlee harbored the highest seroprevalence rate of 33.33% (11/33). At the other locations, the seroprevalence ranged between 4.35% (3/69) and 14.29% (5/35).

### 3.3. TBEV Detection in Ticks

In an attempt to detect and obtain a Belgian TBEV strain, 1983 *Ixodes ricinus* ticks were collected in the areas showing the highest TBEV prevalence in wild boar (Figure 4). These comprised 1515 nymphs, 216 females, 241 males, and 11 larvae. A total of 408 pools were tested for the presence of TBEV RNA by real-time qPCR. No TBEV-RNA-positive ticks were detected.

## 4. Discussion

TBEV is a growing concern in Europe, with increasing alimentary outbreaks and new foci being detected in previously unaffected areas [15,16,52,53,54,55]. Veterinary surveillance is believed to provide more information on the TBEV eco-epidemiological situation in low-prevalence areas than medical surveillance [56,57,58]. Grazing domestic animals (cattle, sheep, and goats) and wild animals (wild boar, roe deer, and red fox) have proven to be suitable sentinels for TBEV detection and surveillance [30,34,38,59,60,61,62,63].

TBEV infection in small ruminants is generally asymptomatic, but TBEV antibodies in these animals have been reported to last for years [64]. No studies to date have assessed TBEV prevalence in these animals in Belgium. We therefore screened a representative sample set of 480 sheep sera from all over Belgium using ELISA to determine the country-wide TBEV seroprevalence in sheep. This ELISA screening resulted in a seroprevalence of 2.71% (0.63% positives and 2.08% borderlines), but only two samples were confirmed as TBEV-positive by the gold-standard seroneutralization test (here performed in PRNT format). The final overall seroprevalence in sheep was therefore 0.42% (CI 95%: 0.11–1.51). It is thus important to point out the different results obtained for some samples according to ELISA and SNT. The ELISA used in this study was a commercial non-competitive indirect ELISA kit that used the horseradish peroxidase protein G conjugate to detect all species’ IgG against the whole inactivated TBEV virus (Neudoerfl strain). Our results suggested that this ELISA kit may have been less flavivirus-specific and more prone to cross-reactions and false-positives compared to the SNT, further emphasizing the indispensability of confirmatory testing. Similar low TBEV seroprevalence rates in sheep were also reported in North-Eastern Germany (0.53%) and Tunisia (0.38%) [33,65]. Higher seroprevalence was, however, found in the Czech Republic (32.5%), Romania (15.02%), Sweden (7.9%), and Hungary (7%) [31,66,67]. These countries are known to be endemic for TBEV, particularly the Czech Republic, while Belgium is currently classified as a country at-risk for TBEV emergence [68]. In addition, the sheep sector in Belgium is small compared to the aforementioned countries and consists mostly of hobbyist farmers keeping a few animals in their backyard [43]. These might be contributing factors to the low TBEV seroprevalence observed in Belgian sheep. The two sheep samples that were confirmed to be TBEV-positive by PRNT were both obtained from the province of Hainaut (Figure 3), suggesting the presence of natural TBEV foci in this province.

Wild boar were suggested to be suitable sentinels to estimate TBEV seroprevalence in endemic areas, since the prevalence in these animals may surpass that of other hosts [38,69,70]. Our serological screening of 831 Flemish wild boar sera by PRNT yielded 142 positive samples. The cross-reactivity of these samples to WNV, USUV, and LIV was analyzed by PRNTs. Twenty-eight samples were finally classified as USUV-positive. This might have been related to the outbreak of USUV in Belgium in 2016 [71,72]. This outbreak occurred in various bird species in the provinces of Antwerp, Limburg, and Flemish Brabant but also in the southern part of Belgium and neighboring countries (France, Germany, and the Netherlands) [71,72]. Although no study has assessed the exposure of wild boar to USUV in Belgium, the geographical spread of USUV in Flanders mostly overlaps with the locations where the 28 wild boar found to be USUV-seropositive in this study were hunted [72]. Thirty-seven of the one hundred and forty-two samples were defined as flavivirus-positive, given that similar titers were obtained for at least two flaviviruses. Seventy-seven of the one hundred and forty-two samples were confirmed to be TBEV-positive, leading to a minimal overall prevalence of 9.27% (CI 95%: 7.48–11.43) in wild boar. A prevalence of 4.20% was previously reported by Roelandt et al. (2016) in Flemish wild boar hunted in 2013 [38]. Our results thus suggest an increase in the prevalence of TBEV in Flemish wild boar (statistically significant, chi^2^: *p* = 0.0118). This suggestion is plausible, since TBEV incidence in Europe has been increasing in recent years [52,73], perhaps because of the increased tick abundance and geographic spread due to climate change [74]. Moreover, the wild boar population in Flanders has increased since the animals’ return in 2006 after half a century of absence [44]. It remains, however, difficult to ascertain the increase in TBEV seroprevalence in the Flemish wild boar population given the differences between the two studies in sample size, screening method, and the Flemish provinces included.

We found that 34/439 wild boar from the province of Limburg were positive for TBEV antibodies. This further emphasizes the presence of TBEV foci in this province. Other studies in wild boar and roe deer have also indicated the presence of TBEV in Limburg [38,40]. This could be related to its location near border areas with the Netherlands, which was thought to be TBEV free until the detection of the virus in ticks collected in 2015. This was followed by reports of human autochthonous cases in 2016 and 2017. Moreover, Rijks et al. (2019) reported new potential TBEV foci located near border areas with Belgium based on roe deer screening for TBEV antibodies [75,76].

Out of the 67 wild boar sera originating from the province of Flemish Brabant, 15 were TBEV-seropositive. The detection of TBEV antibodies in samples from Flemish Brabant has so far only been reported for one roe deer by Tavernier et al. (2015) [40]. Although TBEV-seropositive samples were found in each of the three Flemish provinces, the prevalence of TBEV between these provinces differed. It was the highest in the province of Flemish Brabant (22.39%, CI 95%: 14.06–33.71) compared to the provinces of Antwerp (8.62%, CI 95%: 6.03–12.17) and Limburg (7.74%, CI 95%: 5.59–10.63). This difference was statistically significant (chi^2^: *p* = 0.0005).

TBEV-seropositive wild boar were found in 24 out of the 50 hunting areas, 3 of which (Bree, Dilsen-Stockem, and Maaseik) overlapped with areas where TBEV-seropositive roe deer were found [40]. This could further substantiate the presence of TBEV endemic foci in these spots. Interestingly, a very high TBEV prevalence of 33% was found at Oud-Heverlee. This area contains the Heverlee woods, the Meerdaal forest, and the Egenhoven woods, which form the largest mixed deciduous woods in Flanders. Moreover, red American oak is the predominant tree species in these woods. This could constitute an ideal habitat for *I. ricinus* ticks, which are known to be more abundant in oak forests [77], and also provide appropriate conditions to sustain a rising wild boar population [78]. The high TBEV seroprevalence in wild boar suggests that the risk of acquiring TBEV in this area is higher compared to in other areas, and so it might be advisable to raise awareness among visitors to these forests to avoid tick bites.

Besides providing information on the TBEV seroprevalence in Belgium, our study also aimed to obtain a Belgian TBEV isolate. Therefore, we tested 1983 questing ticks collected from areas that were found to be TBEV hotspots according to the wild boar screening. However, none of the ticks were found to be positive for TBEV. This was in line with the results from Lernout et al. (2019) [79], who also did not detect TBEV in 1599 ticks collected from humans all over Belgium. This was probably related to the low prevalence of TBEV in field-collected ticks, which rarely exceeds 1% even in regions with high human incidence [80], and the fact that TBEV-positive ticks are geographically restricted to small natural foci. This has been illustrated by several studies. For instance, TBEV prevalence was assessed in 8897 questing ticks in known TBEV risk areas in Germany, but none of the 2289 pools were found to be positive. In another study in Switzerland, a total of 62,343 questing ticks were collected at 165 sites throughout the country, and TBEV RNA was detected in only 0.46% of the ticks [80]. Ott et al. (2020) tested a total of 17,893 questing ticks collected in a TBEV high-risk area, and only 7 out of 2228 pools were found to be positive [81]. TBEV detection in ticks seems to be more successful when the microfoci of the virus circulation are identified and when an integrative approach, combining veterinary and medical data, is applied. This was recently illustrated by Alfano et al. (2020), Bournez et al. (2020), and Gonzalez et al. (2022) [16,82,83]. A similar approach involving the collection of ticks in areas where TBEV exposure most likely occurred for the three confirmed autochthonous human cases is therefore recommended for Belgium.

## 5. Conclusions

This study was the first to screen for the presence of TBEV-specific antibodies in Belgian sheep, leading to an estimated seroprevalence of 0.42% (CI 95%: 0.11–1.51). It also provides up-to-date information on the seroprevalence of TBEV in Flemish wild boar, which was estimated to be 9.27% (CI 95%: 7.48–11.43), suggesting an increase in TBEV seroprevalence over the last decade. The high TBEV seroprevalence in wild boar at specific spots indicates the presence of potential TBEV foci in Flanders, although no TBEV Belgian isolate was obtained. A follow-up on TBEV prevalence in wild boar and other sentinel animals is therefore recommended. There is, moreover, a need to increase awareness among the public, veterinarians, and healthcare professionals regarding the potential risk of TBEV emergence in Belgium.

## Figures and Tables

**Table 1:** Number of samples selected from each province.

Province	Selected Samples for the Estimation of TBEV Seroprevalence
Antwerp	37
Limburg	31
East Flanders	126
West Flanders	90
Flemish Brabant	52
**Flanders**	**336**
Walloon Brabant	10
Hainaut	44
Namur	32
Liege	35
Luxembourg	23
**Wallonia**	**144**
**Belgium**	**480**

**Table 2:** The prevalence of tick-borne encephalitis virus prevalence per province in wild boar.

Province	Sampled	TBEV–PRNT Positive	Prevalence (%)
Antwerp	325	28	8.62 (CI 95%: 6.03–12.17)
Limburg	439	34	7.74 (CI 95%: 5.59–10.63)
Flemish Brabant	67	15	22.39 (CI 95%: 14.06–33.71)
**Flanders**	**831**	**77**	**9.27 (CI 95%: 7.48–11.43)**

## Data Availability

The data are included in the manuscript.
